# Biomechanical factors related to occlusal load transfer in removable complete dentures

**DOI:** 10.1007/s10237-014-0642-0

**Published:** 2014-12-20

**Authors:** Jarosław Żmudzki, Grzegorz Chladek, Jacek Kasperski

**Affiliations:** 1 Division of Materials Processing Technology, Institute of Engineering Materials and Biomaterials, Silesian University of Technology, ul. Konarskiego 18a, 44-100 Gliwice, Poland; 2 Department of Prosthetic Dentistry, Medical University of Silesia, pl. Akademicki 17, 41-902 Bytom, Poland

**Keywords:** Complete denture, Pain, Mastication efficiency, Force, Stress

## Abstract

Owing to economic conditions, removable dentures remain popular despite the discomfort and reduced chewing efficiency experienced by most denture wearers. However, there is little evidence to confirm that the level of mucosal load exceeds the pressure pain threshold. This discrepancy stimulated us to review the current state of knowledge on the biomechanics of mastication with complete removable dentures. The loading beneath dentures was analyzed in the context of denture foundation characteristics, salivary lubrication, occlusal forces, and the biomechanics of mastication. The analysis revealed that the interpretation of data collected in vivo is hindered due to the simultaneous overlapping effects of many variables. In turn, problems with determining the pressure beneath a denture and analyzing frictional processes constitute principal limitations of in vitro model studies. Predefined conditions of finite element method simulations should include the effects of oblique mastication forces, simultaneous detachment and sliding of the denture on its foundation, and the stabilizing role of balancing contacts. This review establishes that previous investigations may have failed because of their unsubstantiated assumption that, in a well-working balanced occlusion, force is only exerted perpendicular to the occlusal plane, allowing the denture to sit firmly on its foundation. Recent improvements in the simulation of realistic biomechanical denture behavior raise the possibility of assessing the effects of denture design on the pressures and slides beneath the denture.

## Introduction

Edentulism affects 30–70 % of older people, especially those less prosperous (Müller et al. 2007). Acrylic dentures, also referred to as mucous-borne, soft-tissue-supported, or removable dentures, in which the mucosal membrane forms the prosthetic foundation, are the most commonly used treatment for restoring the dentition.

Unfortunately, the use of removable complete dentures is associated with a high failure rate. Problems with comminution of foods (inefficiency of chewing) and instability of a denture on its foundation (poor retention and stabilization) associated with pain sensations are the most frequently reported causes of treatment failure or denture discomfort (Garrett et al. 1996; Kawano et al. 1996). Studies of the causes of denture discomfort have been undertaken with the aim of improving treatment outcomes. However, the correlation between the clinical assessment of dentures and patients’ judgment ranges from no correlation (Wolff et al. 2003), to weak correlation (Carlsson et al. 1967), to statistically “fair” correlation (Fenlon et al. 2002) to significant correlation (Fenlon et al. 1999a). The results of in vivo studies are difficult to interpret because their assessment of discomfort is based on multiple denture functions summarized by a single score (Carlsson et al. 1967). Even when the different functions are defined and scored separately, the scoring is subject to the simultaneous effects of the many variables in the oral cavity (Fenlon et al. 1999a). Assessment of a denture based on the patient’s perceived comfort could merge biomechanical and completely non-mechanical variables such as perceived individual aesthetics and overall satisfaction (Stober et al. 2012; Matsuda et al. 2014).

Objective evaluation methods of masticatory function in denture wearers have been developed by measuring masticatory performance and efficiency (Garrett et al. 1996; Tokmakci et al. 2013; Yamaga et al. 2013), with electromyographic observation of muscle activity (Garrett et al. 1996; Tokmakci et al. 2013) and masticatory forces and frequency (Lundgren and Laurell 1984; Garrett et al. 1996). In practice, chewing with a denture is evaluated by the wearer to a lesser extent by the efficiency of food comminution, and more as a matter of perceived comfort (Garrett et al. 1996), which is strongly related both to the level of pain in the soft tissue supporting the denture and to salivary lubrication (Wolff et al. 2003; Matsuda et al. 2009). It is noteworthy that more than 80 % of denture wearers suffer from pain resulting from overloading of the soft tissues beneath a denture (Szentpetery et al. 2005). Many biomechanical problems, such as the use of ill-fitting or poorly maintained dentures (Okuma et al. 2004; Fenlon et al. 1999b), can interfere with the biomechanics of dentures under masticatory loads. Denture discomfort has been linked to parafunctional activity; however, it remains only an assumption due to the lack in the study (Kumar 2014) of any reference to the known pain pressure thresholds of the soft tissue that supports dentures (Ogimoto et al. 2002; Tanaka et al. 2004; Ogawa et al. 2004). During functions of speech, facial expressions, swallowing and resting, a denture could cause discomfort simply as a foreign body, even without tissue overloading. Studies using statistical analysis to investigate the influence of clinical variables on patients’ satisfaction (Yamaga et al. 2013) are not the right tool for discovering the mechanisms of load transfer and pain creation.

Some studies related to denture wearing postulate the separation of different aspects of denture biomechanics associated with loads involved in normal wear, speech, swallowing, or mastication (Saber-Sheikh et al. 1999). In clinical studies, basic variables such as occlusal forces, denture displacement, and foundation conditions are barely acknowledged if at all (Garrett et al. 1996; Miyashita et al. 1998; Yamaga et al. 2013; Polyzois et al. 2014; Kumar 2014) making it impossible to discover more than the known principle that denture movement during function is undesirable. Even if the variables could be measured in vivo, the efficiency of mastication is influenced by individual neuromuscular control associated with oral stereognosis (Garrett et al. 1996), which hinder the purely biomechanical phenomenon of mastication load transfer.

Consequently, attempts have been made to examine the functional characteristics of dentures with the aid of physical models and computer analysis with finite element methods (FEM). The outcome of such analyses is determined by predefined conditions of the model and the assessment criteria for the biofunctionality of a denture working on its foundation.

The aim of this paper was to review current evidence regarding occlusal load transfer and pain creation with complete removable soft-tissue-supported dentures, and to establish simulation conditions and physical criteria for the objective assessment of denture comfort during mastication. The hypothesis tested in the review was that pain mechanism investigations are unsuccessful as a result of the unsubstantiated assumption that under well-working balanced occlusion, force is only exerted perpendicular to the occlusal plane, allowing the denture to sit firmly on its foundation.

## Denture foundation

### Criteria of pain and mucous membrane injuries

The mechanical characteristics of the foundation can be described as the cushioning properties (resilience) that endow an ability to bear sustained or cyclic compression. Low resilience is widely postulated as the main determinant of failure in treatment with removable dentures (Kimoto et al. 2007). Most treatment failures are associated with lower dentures whose stabilization conditions are markedly poorer than those of upper dentures (Ozcan et al. 2005; Wolff et al. 2003).

The levels of continuous pressure that can be harmful in terms of mucosal ischemia and development of pressure ulcers vary across the literature. According to some authors, this value is 275 kPa (Kydd et al. 1971). However, another study (Akazawa and Sakurai 2002) showed that pressure ulcers can develop even at pressures of 50–150 kPa; such pressures were associated with a significant (up to 15 %) decrease in blood perfusion acting for at least 20 seconds. Another researcher (Maruo et al. 2010) considered pressures of 67.5–90.8 kPa to be harmful to mucosal tissues. One study (Maruo et al. 2010) revealed that exposure of soft tissues to pressures of a magnitude detrimental to blood flow can induce the atrophy of alveolar processes. The perfusion of tissues turned out to be significantly better in the case of cyclic loads (Akazawa and Sakurai 2002; Okada et al. 2010). Mechanical stimulation of the palatal mucosa with a pressure of approximately 50 kPa repeated over a period of 12 min (960 cycles) may induce an increase in the blood flow (Ono 1990; Okada et al. 2010). However, this compression magnitude (Okada et al. 2010) does not fit with the expected mastication pressures. Denture wearers often masticate despite their pain. Therefore, higher pressures, corresponding to the pressure pain threshold (PPT), should be defined as harmful in the case of tissues exposed to cyclic loads. However, the values of cyclic pressures that are traumatic for the masticatory mucosa are not known.

The PPT of the mucosal membrane is the most important property of the denture foundation (Ogimoto et al. 2002; Tanaka et al. 2004; Ogawa et al. 2004). Similar values relating to painful pressure for the masticatory mucosa have been documented by several researchers: 370–1,800 kPa (Abe et al. 1998), 399–1,873 kPa (Ogawa et al. 2004) 270–1,720 kPa (Isobe et al. 2013), and 686–1,372 kPa (Miyashita 1969). Recordings of the perceived intensity of pain on a 0–50–100 scale were undertaken by Naganawa (2013), where 50 is defined as “just barely painful” and 100 is defined as “the most pain imaginable.” The range of 50–70 is equated to a pressure of approximately 1,400–2,700 kPa applied for 2 s. The pressure values listed in this paragraph were estimated using the indenter tip and force reported in each study in order to compare the studies.

Mechanical interference to the denture can lead to mucosal injuries; this type of complication is quite frequent, as it can affect up to 15–20 % of denture wearers (Jainkittivong et al. 2002). It is noteworthy, however, that the presence of pain and the prevalence of mucosal injuries did not turn out to be significantly associated (Ozcan et al. 2005). The PPT can decrease by 40 % as a result of long-term denture wearing (Tanaka et al. 2004), although this is only related to the palatal mucosa; alveolar soft tissues have similar PPTs for edentulous and dentate subjects. The average value of PPT in the edentate state in the area of the premolar teeth, which is exposed to the greatest occlusal forces, amounts to approximately 630 kPa (Ogawa et al. 2004). The 2-fold higher PPT for the edentulous palatal mucosa over the alveolus (3- to 4.65-fold higher in dentate subjects) is explained by the lack of innervation in the palatal mucosa rather than the decreased tissue thickness in the alveolar mucosa (Tanaka et al. 2004; Ogimoto et al. 2002). Higher bite forces correlate with a lower PPT, particularly in the posterior region, and this effect is stronger in the mandible than in the maxilla (Tanaka et al. 2004).

### Mucous membrane material models

Elasticity and creep play crucial roles in the process of soft tissue deformation. Viscoelastic behavior is associated with fluid interchange with surrounding unstressed mucoperiosteum and the displacement of large polymer molecules of the soft connective tissues (Scapino 1967). Hydrated porcine oral mucosa samples (10 mm in diameter and 1.5 mm thick) that were subjected to 5 min of 0.32 kPa static load experienced a 3-fold increase in strain to 3.2 %, and in the moment of unloading exhibited 1 % elastic instantaneous recovery (Lacoste-Ferré et al. 2011). Despite the greater precision achieved when measuring non-living samples, the results are influenced by a lack of body fluid perfusion.

These phenomena can be simulated by the movement of a damper embedded in a viscous fluid and connected in parallel to a spring (Voigt model). In living tissue, the “weaker” the spring, the greater the deformation associated with the intra-tissue viscous flow and the worse the ability to shape recovery after unloading. However, a larger number of dampers with a given viscosity of fluid $(\upeta )$, springs with a given elasticity (E) (as in the Voigt and Maxwell models), and various different arrangements (i.e., in series and in parallel) need to be introduced to the model in order to simulate the true behavior of soft tissues; a four-element model can be used. The modulus of elasticity and viscous coefficient in a parallel model (Maxwell) measured 1.1 MPa and 250 MPa*s, and in serial model (Voigt), they measured 1.2 MPa and 18 MPa*s (Tanaka 1973). Somewhat different values have also been found (Hayakawa et al. 1994): 0.36–0.59 MPa and 667–689 MPa*s for elements of a parallel model; and 1.41 MPa and 56–63 MPa*s for elements of a serial model.

However, the deformations and strains under indentation tests are quite different from those beneath a denture saddle owing to the constraints of deformation of nearly incompressible soft tissue. The mechanical characteristics of mucosa become markedly stiffer when measured under surfaces with an increased interfacial coverage (Wills and Manderson 1977). A higher gradient of deformation is evident around small-diameter indenters that fall easily and deeply into the tissue (Isobe et al. 2013; Wakabayashi and Suzuki 2013; Yoshida et al. 1999). For instance, a cylindrical rod 1.3 mm in diameter indents 0.4 mm into the tissue under a relatively low pressure of 37.6 kPa (Wakabayashi and Suzuki 2013). However, beneath a denture saddle, despite the higher pressure applied, the compressed zone is markedly larger and the uncompressed tissues are more distant, even when the denture is tilted during mastication (Żmudzki et al. 2012). These implications are emphasized in the section about loading of the denture foundation during mastication.

Indentation experiments, which impose a high gradient of deformation over periods longer than the chewing cycle, create conditions that force the soft tissue to undergo more viscous flow than would occur during mastication. Although the conditions of indentation experiments favor fluid interchange, the viscous flow increased the instantaneous displacement from 0.36 mm at 0.1 s to 0.40 mm after 10 s loading, an increase of only 10 % (Wakabayashi and Suzuki 2013). Another study (Yoshida et al. 1999) found that strain recovery before a subsequent masticatory load (i.e., when mucosal support is important) worsens with age. The authors reported the displacement level and duration, and the indenter diameter (3.1 mm); however, the pressure level cannot be calculated because the force values were not disclosed. The highest indenter displacements (0.3 mm) were recovered to the level of 80 % at approximately 0.81 s and 0.44 s in the two oldest subjects. The average time for mucosa displaced 0.3 mm to recover to 80 % in subjects beyond 50 years of age was in the range of 0.5–0.6 s (on the basis of graphs, because the authors did not tabularize values). Although the statistics indicate that mucosa worsens with age, it is hard to agree with generalization of the results in relation to insufficient mucosal recovery in older persons. The author (Yoshida et al. 1999) assumed a relatively short chewing cycle period of 0.5 s as the criterion, and 0.3 mm of displacement as the initial level, regardless of the mucosa resilience, which was not measured. The resilience measured with the shear modulus in the work of Wakabayashi and Suzuki (2013) was about one-third less $(\hbox {G}_{0}=0.07~\hbox {MPa})$ for thinner soft tissue than for the thickest soft tissue $(\hbox {G}_{0}=0.095~\hbox {MPa})$. This elastic variability was assumed to explain the visible tendency for higher viscous behavior under 10 s continuous loading for thinner soft tissue compared with thicker soft tissue, although the possibility of a high strain gradient was not taken into consideration. Because it is impossible to distinguish which variable (elasticity, thickness, strain gradient) had a greater influence on the viscous flow, it is necessary to investigate the significance of the strain gradient, especially given that the recovery rate after 20 s unloading was similar for all the subjects. Additionally, the PPT increases almost linearly with increases in the loading rate (Ogimoto et al. 2002). To minimize intraindividual variability, a constant pressure increase of 156 kPa per second was assumed by Ogimoto (Ogimoto et al. 2002; Ogawa et al. 2004). However, because PPT is achieved during mastication in practice, higher pressures are generated in a shorter time than this assumption. In fact, studies by Yoshida et al. (1999) and Wakabayashi and Suzuki (2013) agree that the mucosa recovers by about 80–90 % during each chewing cycle.

Elasticity and creep also are characterized in dynamic compression experiments under a chewing frequency of 1 Hz to determine the complex modulus E* (G* under shear), consisting of the storage modulus $\hbox {E}^{\prime }$, which is equivalent to Young’s modulus, and the loss modulus $\hbox {E}^{\prime \prime }$, which is associated with the dissipative (viscous) component. The damping factor (the amount of energy dissipated as heat through a cycle of deformation) is also denoted as loss tangent (tan $\delta$), i.e., $\hbox {E}^{\prime \prime }/\hbox {E}^{\prime }$ (Saber-Sheikh et al. 1999; Lacoste-Ferré et al. 2011). Dried samples under compression had a higher storage modulus $\hbox {E}^{\prime }$ of 3–4 MPa, while the elasticity decreased with hydration to 0.1–0.2 MPa (Lacoste-Ferré et al. 2011). Some stiffening of the hydrated oral mucosa was observed during the second series of 20- min cycling compression, and a residual strain of 0.25 % was measured during the static creep experiment. The deformation of samples did not directly correspond to the creep of living tissue because of the process of cutting and the lack of body fluid perfusion. Dynamic cycling tests were made under a relatively small strain of 0.01–0.2 %; thus, the measured modulus is lower with reference to the mastication loads due to the hyperelastic behavior and the surface “softness” of the rough (i.e., with papillae) and moistened biological tissue. The low value of the elastic modulus noted by Lacoste-Ferré et al. 2011 corresponds with the shear modulus reported by Wakabayashi and Suzuki (2013), because in both cases the initial rigidity is acquired. In an analysis of denture soft liners (Lacoste-Ferré et al. 2011), the authors assumed a higher value of 2.72 MPa for the storage modulus ($\hbox {E}^{'}$) of the masticatory mucosa on the basis of a previous in vivo investigation (Inoue et al. 1985), although the value of load during masticatory mucosa compression in the source test was not clearly stated.

However, usually one strives for a simplification of the mathematical processes taking place in tissues during FEM simulation of more complex three-dimensional systems, such as denture biomechanics. In a situation with a convergence problem caused by simultaneous large body motion and nonlinear deformation, it is necessary to decide which issue is less important in in vivo biomechanical behavior. Although mucosa exhibits nonlinear and time-dependent characteristics, hyperelastic strain energy density functions in a simplified form based on a one-dimensional test (Fung 1993) can be applicable, but linear isotropic simplification is much more robust. When the displacements are mainly a result of denture movement and associated contact phenomena, although the mucosa experiences deformation beyond the theoretical applicability of the linear range, an approximation of the material nonlinearities avoids the difficulties with finite element analysis because the excessively distorted elements occur at the “initial softness” of the hyperelastic curve.

The modulus of elasticity of mucosa measured with ultrasound is within the range of 0.91–5.93 MPa (Isobe et al. 2013), and there is broad agreement with this among mechanical measurements taken 0.37–5.80 MPa (Tanaka 1973), 0.41–2.67 MPa (Nakashima 1975), 0.66–4.36 MPa (Inoue et al. 1985), and 2.75–5.03 MPa (Józefowicz 1970). The modulus of elasticity cannot be estimated from the thickness of the mucosa due to a lack of correlation between them (Isobe et al. 2013; Wakabayashi and Suzuki 2013). Because linearization of the hyperelastic curve can generate many different elastic modulus values, the transversal line must lead appropriately to the stress level accompanying mastication and the curve must be taken under the exact load rate. In these conditions, tissue which has a greater ability to undergo elastic deformation and rapid recovery allows the pressure to be distributed more evenly. There are superior cushioning properties in the larger area under the stress–strain curve (and transversal line); this can be ascertained by palpation and evaluated as the more resilient mucosa. However, it must be kept in mind that linearization of the hyperelastic curve overestimates stresses in low-loaded tissue and underestimates stresses in high-loaded tissue. In addition to the studies on the characteristics of denture-supporting tissue, there is evidence (Wolff et al. 2003) that shows a lack of significant correlation between the resilience of mucosa and denture satisfaction when salivation is impaired.

## Salivary lubrication

### Salivary properties in biofilm formation

The role of salivary properties is crucial as a potential determinant of the functional performance of dentures. Impaired salivation causes chewing and speaking discomfort, as well as pain in the denture bearing tissues in complete denture wearers (Wolff et al. 2003). Previous studies centered on the amount of secreted saliva, the thickness of the salivary layer, and its density and viscosity. The thickness of the salivary layer that covers oral tissues can range from a few to more than $100~\upmu \hbox {m}$ (Wolff and Kleinberg 1998). While the amount of secreted saliva decreases with the duration of chewing, it is not affected by chewing frequency (Dong et al. 1995). Bite force correlates with salivary flow (Yeh et al. 2000). Stimulated and unstimulated average salivary flow rate increased from 0.45 and 0.06 mL/min, respectively, before replacement dentures to 0.75 and 0.10 mL/min, after fitting of new complete replacement dentures (Matsuda et al. 2009). Matsuda et al. (2009) stated that salivary flow rate after denture replacement increases with improvement in the maximal occlusal force.

Saliva changes the surface of the mucosa from hydrophobic to hydrophilic as a result of the selective adsorption of salivary molecules. The value of $50.5~\pm ~2.4^{\circ }$ for the water contact angle of a saliva-lubricated tongue suggests that its hydrophilicity is greater than that of a non-lubricated tongue, for which the water contact angle is $77.3~\pm ~4.1^{\circ }$ (Ranc et al. 2006). The effect of a salivary coating on surface free energy and wettability was investigated by Sipahi et al. (2001). The authors concluded that more wettable materials, such as light-cured and heat-cured acrylic resins, are a good choice for clinical use, because more wettable materials can improve denture retention (Sipahi et al. 2001). It should be noted that greater hydrophilicity can promote yeast adhesion to dental materials (Kang et al. 2013). Although this correlation is not always clear (Chladek et al. 2014), a biofilm formation study is needed that not only takes into consideration the adherence of microorganisms on different denture surfaces, but also emphasizes how the viscosity and lubrication properties (Ranc et al. 2006) influence denture sliding (Żmudzki et al. 2012b) rather than denture retention because of the weak correlation of denture retention with chewing efficiency (Müller et al. 2002).

Saliva, like a many biological fluids, shows abnormality of viscosity with shear rate, typical for non-Newtonian fluids. The lubrication properties of saliva result from mucins, statherin, and proline-rich proteins (Hahn Berg et al. 2004), although statherin (Proctor et al. 2005) and mucins (van der Reijden 1993) are more prominent. One study (Proctor et al. 2005) emphasizes the amphiphatic nature of statherin as a source of its lubrication properties. Another study (van der Reijden 1993) showed that mucin separated from saliva exhibits similar viscoelastic behavior to saliva; thus, the authors conclude that mucin, because of its marked elasticity and low viscosity, is important in the creation and maintenance of the coating on mucosa. Differences in the rheological properties of saliva are mostly explained as a result of the mixed internal structure of mucin and subsequently as a dissimilarity in the saliva concentration (van der Reijden 1993).

### Sliding on surface covered by saliva

An engineering-style mechanical analysis of contact interaction is necessary to simplify the complex phenomenon of resistance to motion provided by the saliva coating. The friction coefficient is also used as a measure of salivary lubrication, but the values of this parameter are highly variable. In one study, the dynamic friction coefficient determined at a velocity of 0.5 mm/s and a normal load of 0.1 N amounted to 0.16 $(\pm 0.03)$ for piglet tongues covered with human saliva and 0.25 $(\pm ~0.03)$ for non-lubricated piglet tongues (Ranc et al. 2006). It is noteworthy that stimulated saliva secreted during chewing shows inferior lubricating properties (Prinz et al. 2007), resulting from its lower viscosity (greater dilution), as compared with non-stimulated saliva. The coefficient of friction decreased from 0.25 to 0.1 for unstimulated saliva, and from 0.33 to 0.16 for stimulated saliva, at speeds ranging from 0.7 to 9.8 mm/s (Prinz et al. 2007). The decrease in the coefficient of friction for both types of saliva was explained by probable hydrodynamic effects because in the slower speed range (0.1–0.5 mm/s), the friction was constant, suggesting a boundary lubrication regime. A decrease in the friction coefficient, proportional to an increase in normal load from 0.34 to 2.20 N, is explained in terms of possible superficial deformation of micro-irregularities in the tissue. However, markedly higher values of the coefficient, up to 0.45, were reported by de Wijk and Prinz (2005). Important evidence originates from Bongaerts et al. (2007) who, despite studying saliva samples from only two persons, showed that the friction coefficient is to a large extent determined by the degree of salivary hydration (or dehydration). Whereas the friction coefficient of fresh hydrated saliva amounted to 0.02–0.06, the analogous parameter of the dehydrated material reached 2.8–3.0, indicating strong adhesion (Bongaerts et al. 2007). Despite many investigations, there is uncertainty related to the frictional characteristics of the tribo pair of denture base/mucosa, because it is not known that in vitro studies accurately represent realistic soft contact in terms of compliance, surface roughness, and surface chemistry (Bongaerts et al. 2007). Bongaerts et al. (2007) suggested that the much higher friction of hydrated saliva reported earlier by de Wijk and Prinz (2005) may be the result of higher surface roughness, although it seems that the main reason should relate to adhesion phenomena and surface compliance, as well as bulk deformation generating resistance dependent on interface shape during motion. Thus, further extensive experiments with motion resistance and denture slide are required to assess denture materials and masticatory mucosa in regard to interfacial—and also bulk—properties and salivary wetting.

## Biting force and the biomechanics of mastication

### Food comminution with complete denture

Up to 36 % of variance in chewing efficiency with normal dentition is explained by the maximum biting (occlusal) force generated by molar teeth and only 9 % by the characteristics of the occlusal surface (Lujan-Climent et al. 2008). The situation is completely different in the case of removable dentures, as the ability of wearers to comminute foods is modulated by a number of variables.

A masticatory cycle of removable dentures approximates 1.3 $(\pm ~0.20)$ Hz (Veyrune et al. 2007). It is noteworthy that the average period of occlusal pressure (from start to finish) is quoted as 0.110–0.169 s (Okuma et al. 2004), compared with the commonly accepted time of 0.5 s for half the chewing cycle. Therefore, the duration of occlusal pressure is shorter than the chewing cycle, which leaves a longer time for recovery from mucosal deformation than the relatively short time of half a chewing cycle (see Sect. 2). Movements of the mandible and occlusal forces reflect the activity of the mandibular muscles. While the amplitude of muscular impulses in removable denture wearers is twice as high as in persons with normal dentition, the two groups do not differ significantly in terms of the impulse duration (Slagter et al. 1993). Furthermore, neither the status of the dentition nor the texture of food seem to exert a significant effect on the rhythm of mandibular movements. Finally, the amplitude of the muscular impulse is only weakly correlated with the degree of food comminution. Consequently, functional assessment of dentures should not be based on the activity of muscles, owing to a lack of evident correlation with chewing efficiency (Hayakawa et al. 2000). Muscle activity is greater after denture delivery and decreases during adaptation (Tokmakci et al. 2013). Even if there is evidence for using the analysis of muscular activity (Tokmakci et al. 2013) and occlusion period (Okuma et al. 2004) in evaluating adaptation to a denture, other essential biomechanical variables are not taken into consideration in this method.

Settlement of a denture on its foundation can be analyzed as a resistance to vertical detachment (defined as retention) and horizontal load (stabilization). Retention is defined as the force required for moving a denture from its foundation in the direction opposite to insertion. The average retention and stabilization forces for lower dentures amount to 0.8–3.0 and 2.8–7.2 N, respectively (Burns et al. 1995). Higher levels of retention (3.9–4.7 N) have been documented for upper dentures (Tallgren 1959). It is of note, however, that both retention and stabilization, albeit considered as the principal determinants of functional comfort, are in fact weakly correlated with chewing efficiency (Müller et al. 2002). The lesser role of retention and stabilization during chewing results from the fact that the distribution of forces affecting the denture during mastication is quite different, even if resistance against a lateral rotating force by finger pressure on the molar section is scored in the stability test.

### Occlusal loads

Occlusal forces in the range of 65–110 N, generated at the level of premolar and molar teeth, has been shown to be sufficient for comminution of most foods (Ogata and Satoh 1995). The incisor teeth of removable dentures are oriented in a way that prevents their involvement in food comminution. Even loads at the incisal region as low as 10 N cause elevation of the denture flanges and loss of stability, although load-bearing ability increases approximately 20 % when denture adhesives are used (Polyzois et al. 2014). Kalra et al. (2012) reports even greater increases in load-bearing ability (from 9.8 to 29.3–42.9 N) in dentures rated as “poor” depending on the type of adhesive used. Detailed information on the distribution of occlusal forces on the masticatory surfaces of teeth can be obtained with the aid of special occlusion foils (Alkan et al. 2006) that allow pressure to be measured as the degree of coloring in an elementary unit of a square of 0.25 mm. Although the foil is thin in comparison with other methods, and it seems to be an efficient and economical way to record pressure patterns, it is hard to agree that its flexibility is sufficient to permit natural occlusion (Alkan et al. 2006). In one experiment using this method (Lee et al. 2008), the average maximum occlusal force amounted to 122 N (range 79–461 N). The occlusal forces determined in another study (Tanaka et al. 2004) ranged from 28.2 to 166.5 N, with mean value of 97.1 N and a standard deviation of 46.3 N. The maximum occlusal force increased from 84 to 165 N after replacement of old dentures and 2–3 adjustments for 22 subjects who had been wearing dentures for more than 5 years (Matsuda et al. 2009).

### Biomechanics of mastication

It should be noted, however, that unlike normal dentition, denture chewing efficiency is only weakly correlated with maximum occlusal force (Fontijn-Tekamp et al. 2000). The maximum occlusal force at denture dislodgement is measured under stable conditions, in the so-called best biting position (Yeh et al. 2000; Matsuda et al. 2009; Polyzois et al. 2014; Matsuda et al. 2014); hence, it is not a relevant measure of clinical function (Koyano et al. 2012). The forces affecting the denture during the measurements do not reflect the stabilization associated with usual mastication and the pain sensations beneath the denture. The method of maximal occlusal force measurement has been adopted because under unilateral loads, dentures dislodge excessively rapidly (Yeh et al. 2000; Matsuda et al. 2009), in exactly the same way as during mastication without balanced contacts.

Chewing efficiency is assessed by the degree of food particle comminution (Demers et al. 1996). In many studies, structural characteristics of dentures were analyzed on the basis of changes in occlusal forces, muscle activity, masticatory cycles, and mandibular displacement (Karlsson and Carlsson 1990; Khamis and Zaki 1997). Although a certain set of structural characteristics of a denture exerts some effect on chewing efficiency under given foundation conditions, significant correlations were documented solely in the case of lingualized occlusion.

During chewing, a lower denture has to be supported by the upper one by means of the so-called balancing contacts; the lack of balancing contacts results in dislodgement. The effect of balancing contacts is a prerequisite for chewing efficiency (Kobayashi 1989). Food comminution results from crushing particles over a relatively short distance, no greater than 2–3 mm, with the involvement of lateral, but not anteroposterior, pounding movements of the mandible. However, most analyzed models are too simplified, as only the effect of isolated vertical force is assumed (Kumar 2014; Takayama et al. 2011; Kawano et al. 1993). This idealization may result from limitations in the measurement methods, as usually only the vertical component of force is measured, but the horizontal component of force is also present as determined by cusp shapes. The argument against simplification is that, as a result of an occlusal contact on the balancing side, the masticatory force is only shifted toward the interior of the arch and not to the center (Ogata and Satoh 1995). Mastication forces are not generated on teeth through direct contact, but through the food; this is another explanation for the weak dependence of mastication efficiency on the quality of the denture occlusion (Koyano et al. 2012). The force direction is dependent on the geometry and consistency of the food fragment and can be more oblique than the 30$^{\circ }$–35$^{\circ }$ found in the inclination of artificial tooth cusps. Therefore, lateral deviation (up to $45^{\circ }$) of mastication force from a vertical plane is postulated as the most appropriate, because in engineering the most unfavorable loads should be taken into consideration (Kenney and Richards 1998; Żmudzki et al. 2012; Liu et al. 2013; Shim and Watts 2000; Akan et al. 2010), although the investigations into the force angulations causing elevation of the non-loaded flange are useful. According to the available data, the mobility of dentures during chewing can be successfully determined in situ, i.e., in the oral cavity. Balancing contacts were revealed to precede occlusal development of occlusal pressures on the working side (Kobayashi 1989). However, a relatively large displacement is observed during the mastication phase of chewing despite optimal adjustment of dentures to their foundations and functional balancing (Miyashita et al. 1998; Rendell et al. 1995). In a mechanical context, a “contact” is also a force. The use of the term balancing “contact” reflects the predominance of the kinematic approach to a denture’s biostatics, which is usually considered as an equivalent of its mobility. According to one study (Chong 1983), the displacement at the working and balancing side can be as high as 1.4 and 1.6 mm, respectively. The balancing side shows a marked tendency to detachment and sliding on its foundation (Miyashita et al. 1998), and importantly, displacement does not occur during opening, but under load. Miyashita et al. (1998) documented a tendency for a loss in chewing efficiency with hard foods (e.g., carrots) compared with softer foods (e.g., fish paste) as a result of an increase in the anteroposterior mobility of a denture. The large displacement of dentures explains the inconsistencies and weak correlations between maximal occlusal force (measured in a stable position) and masticatory efficiency. Miyashita et al. (1998) shows that the denture’s balance is achieved in a horizontally displaced position in relation to the foundation, but there is no evidence that the balancing flange sits on the foundation as the result of the balancing contact. However, it cannot be ruled out during the last stage of mastication without further in vivo investigations.

## Loading of the denture foundation under mastication forces

### Experimental in vivo measurements and clinical facts

Soft tissue denture foundation loadings are commonly considered a cause of pressure ulcers. However, in practice, they usually cause abrasions, rather than ulcers (Mackenzie and Ettinger 1975; Martínez Díaz-Canel and García-Pola Vallejo 2002). Mobility of the denture on its foundation is a potential source of mucosal injury and discomfort, especially while salivation is impaired (Rendell et al. 1995). The degree of displacement is greater if the denture is not appropriately adjusted to its foundation (Rendell et al. 1995). However, at present, we lack experimental methods that could be used for distinguishing between displacement resulting from the deformation of a soft foundation and those resulting from the detachment and sliding of the denture.

Some researchers have mistakenly taken denture displacement to relate only to the deformation of the denture-supporting tissue (Compagnoni et al. 2003). The extent of mucosal deformation resulting from pressure exerted by a denture saddle amounts to 0.1–0.3 mm (Kramer 2004), which corresponds to a 5–20 % strain, depending on mucosal stiffness (Wakabayashi and Suzuki 2013). Displacements that are greater than or similar to the mucosal thickness result from the combined effect of displacement of the denture on its foundation and mucosal deformation. The extent of mucosal deformation caused by a denture saddle should not exceed the thickness of the mucosal membrane. Severe deformation, involving the entire thickness of the mucosal membrane, may result from loading a small area that is particularly susceptible to shape (distortional) deformation, e.g., during the determination of resilience with the aid of an indenter (Wakabayashi and Suzuki 2013). It seems that Compagnoni et al. (2003) assumed that denture displacement could be measured as a deformation of the mucosa because of the significant level of deformation observed in the mucosa during indentation experiments. Kinesiographic methods of determining denture displacement on its foundation are not suitable for examining mucosal deformation, because the recorded values (0.1–0.3 mm) fit within the range of the measurement bias.

Dentures that promote equal distribution of the soft tissue foundation loading and mastication comfort (Garrett et al. 1996) are more acceptable, even if their use is not reflected by a measurable improvement in chewing efficiency. Pain and discomfort experienced beneath a denture during chewing stimulated research on the phenomenon of masticatory load transfer. The experimental technique enabling the assessment of mucosal load beneath a denture is based on the determination of pressure with the aid of various sensors (Ohguri et al. 1999; Taguchi et al. 2001). However, only local measurements can be taken this way, and thus, the global distribution of pressure needs to be averaged. Sensors can be constructed that enable measurements to be taken solely on the slopes of the alveolar processes, rather than on their crests. Recently, attempts were undertaken to determine the distribution of pressure on the entire surface of the slopes with the aid of special sensor sheets (Kubo et al. 2009). However, only the pressure under a short segment of a partial denture saddle supported by evenly shaped convex alveolar processes has been determined to date (Kubo et al. 2009). Unfortunately, the excessive stiffness of the sensor sheet means that it cannot be optimally adjusted to longer segments of the processes forming the alveolar arch, or where the ridges are atrophied or irregular in shape. Furthermore, the presence of the sensor sheet at the denture–foundation interface disturbs the status of soft tissue loading, especially in the case of sliding, which is the principal risk factor for mucosal abrasion. Finally, the above-mentioned experimental techniques cannot be used for determining deep tissue loading or abrasive effects.

### Modelling study

Finite element analysis is an established tool for the assessment of the global mucosal membrane load. The equivalent stresses on a mucosal surface, determined according to the Huber–Mises (H–M) hypothesis, constitute the only interpretation criterion of the numeric analyses, providing information on the degree of distortional deformation of tissues (Kawano et al. 1990, 1993; Sato et al. 2000; Takayama et al. 2001; Kawasaki et al. 2001; Ateş et al. 2006). Surprisingly, however, the pressures beneath a denture are usually not analyzed using the finite element method. According to the achievements in ulcer prevention techniques (Oomens et al. 2003; Ragan et al. 2002), the above-mentioned determinants of loading, i.e., shear and pressure, should not be analyzed as separate criteria, as this can lead to misinterpretation of findings. Separate areas of elevated pressure and shear were identified in the mucosa beneath a denture as well (Kasperski et al. 2010), but the applicability of this finding is limited due to the simplified model conditions that assume an ideal denture foundation.

Few authors have considered the abrasive processes beneath a denture as a criterion for the traumatic effect on the mucosal foundation. Takayama et al. (2011) simulated the sliding of a denture loaded with vertical forces, but they eventually did not report on the contact stresses beneath the denture. They reported the denture movement as neither actual nor functional, although equivalent to the clinical examination of the stability of dentures under bilateral and unilateral pressure over the occlusal surface (Gerber 1974). Small denture movements resulted from the displaceability of the mucosa, and small slides were associated with clenching or swallowing; hence, the lack of realistic slides accompanying mastication provides no basis for evaluating (Takayama et al. 2011) the selection and arrangement of artificial teeth as desirable and with good adaptation to the ridge. Another study (Kawasaki et al. 2001), based on a numeric simulation of a denture slide on a mucosal surface, showed that the risk of abrasion increases when there is atrophy of the edentulous alveolar ridge in the anterior mandibular segment; as a result, the denture slides down anteriorly on its slope foundation. However, the high incidence of abrasions beneath dentures suggests that these injuries are not necessarily associated with the characteristic slope shape of the foundation. According to clinicians, they rather result from the mobility of denture and the degree of stabilization achieved by balancing contacts. Mathematical modelling of the effect of balancing contacts on the stabilization of dentures was studied by Lü et al. (2010); unfortunately, it was conducted with assumed complete adherence of denture to the foundation.

**Fig. 1:**
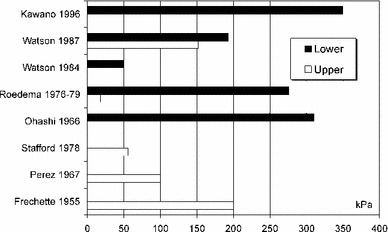
Pressures on mucosal membrane beneath lower and upper denture determined in vivo (Frechette 1955a, b; Perez 1967; Stafford 1978; Roedema 1976, 1979; Ohashi et al. 1966; Watson and Abdul Wahab 1984; Watson and Huggett 1987; Kawano et al. 1996)

**Fig. 2:**
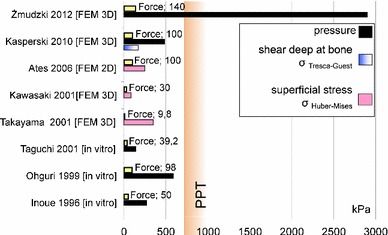
Results of physical and FEM investigations of bearing the occlusal force (in Newtons) in the criteria of stresses beneath denture: pressure (compression stress), shear stress in deep at bone (Tresca–Guest stress), superficial stress (Huber–Mises stress) (Taguchi et al. 2001; Takayama et al. 2001; Kawasaki et al. 2001; Ateş et al. 2006; Inoue et al. 1996; Ohguri et al. 1999; Kasperski et al. 2010)

The determined in vivo values of pressure beneath complete dentures are presented in Fig. 1, and the values of load determined in vitro are shown in Fig. 2 (Taguchi et al. 2001; Takayama et al. 2001; Kawasaki et al. 2001; Ateş et al. 2006; Frechette 1955a, b; Perez 1967; Stafford 1978; Roedema 1976, 1979; Ohashi et al. 1966; Watson and Abdul Wahab 1984; Watson and Huggett 1987; Kawano et al. 1996; Inoue et al. 1996; Ohguri et al. 1999; Kasperski et al. 2010). Owing to advances in the methodology of FEM modelling and the improved simulation of mechanical characteristics of the mucosal membrane (Kawano et al. 1990, 1993), it was documented that, for revealing the mechanism of pain, the implementation of the rheological model of the mucosa is not conclusive. Its influence on the stress level was less than 10 %, while shifting of the vertical occlusal force from the palatal to buccal side caused an increase in stress of more than 40 % (percentages are used here because the increase in the maximal stress at the alveolar crest from 300 kPa at 0.1 s to 320 kPa at 3 s after loading is calculated using plane stress analysis and without data about the model thickness). Notably, the values for pressure that were registered beneath dentures are markedly lower than the PPT (Tanaka et al. 2004; Ogawa et al. 2004; Miyashita 1969), which is inconsistent with the pain and discomfort reported by the vast majority of denture wearers. This discrepancy seems to be associated with inappropriate simulation of the loading and supporting conditions of a denture. Namely, the measurements are taken under overly stable conditions, with the working denture being evenly supported by its foundation (Frechette 1955a). Under such conditions, one can hardly simulate the typical elevation of a denture flange on the balancing side; in contrast, the pressures recorded under the balancing flange, being absent in vivo, are higher than on the working side.

### FEA key futures

Żmudzki et al. (2012) used FEM to simulate the results of mastication with a denture placed on an unfavorable foundation, i.e., tilting of a denture under an oblique mastication force and a decreased area of support, with subsequent stabilization due to the balancing contacts (Miyashita et al. 1998; Rendell et al. 1995; Chong 1983). The compression beneath the denture is transferred in the form of a large slide, which is in accordance with common clinical observations of frictional mucous membrane sores and/or the impact of reduced salivation on denture discomfort and chewing deficiency (Wolff et al. 2003). Mastication load transfer was previously only recognized for linear elastic mucosa (Żmudzki et al. 2012), and further study on the influence of viscous flow is necessary, although the prevalent impact of denture destabilization has been revealed (an order of magnitude) in comparison with the slight influence (several percent) of viscoelasticity, which was previously considered to be the most important factor (Kawano et al. 1990, 1993). Compression notably exceeded average PPT (by 4.7 times) and only approximately 30 N oblique occlusal force can be borne at the PPT, which is consistent with a common decrease in chewing efficiency in analyzed cases of unfavorable foundation conditions (Slagter et al. 1993). The pressure beneath the same denture (Żmudzki et al. 2012) that was loaded in a stable non-realistic manner (with a vertical force of 100 N) was lower than the PPT, although extremely unfavorable foundations were chosen for the analysis. The force on the balancing contacts is significant and decreases from 25.9 to 20.9 N when the initial distance to the upper denture surface increases from 0.1 to 1.0 mm (with “delayed” balancing contact). The compression and sliding distance on the mucous membrane increases when the balancing contact is delayed.

The augmented multiplier Lagrangian method may be used (Żmudzki et al. 2012, 2013) in contact calculations with an implemented linear friction model and neglecting adhesive forces incomparably lower than mastication loads. Using such a penalty function, in cases where there is a marked difference in elasticity between contacting bodies, contact stiffness and allowable penetration must be adjusted to achieve convergence (Żmudzki 2011; Żmudzki et al. 2013b). Żmudzki et al. (2012) assumed an average friction coefficient of 0.16; thus, the effect of friction variability on mucosa compression is not known, although its influence on the biomechanics of a removable single implant-retained denture was marked (Żmudzki et al. 2012b). Implant load increased from 45.9 to 81.5 N when the change in the friction coefficient reached between 0.5 and 0.02. The lower friction resulted in an increase in the load on the implant, which could to a certain degree substitute for the balancing contact (Żmudzki et al. 2013).

It must be emphasized that a more dense meshing does not imply quality enhancement. Even when tied contact is taken into account and the mesh is coherent at the interface, the calculated stress increases to non-realistic values at the singularity point as it was demonstrated in the bone tissue margin around a dental implant neck (Żmudzki et al. 2008). In a similar manner, an increase in mesh density at the contacting interface resulted in a concentration of the stresses around the unfavorably positioned contact elements (having normals not aligned with opposite contact elements) or in a lack of convergence (Zienkiewicz and Taylor 2005). The influence of mesh density on the contact stress values between two cylinders, with a modulus of elasticity mimicking mucosa and denture, was calculated by FEM and verified with analytical Hertz formula in Żmudzki’s work (2013b).

## Clinical implications

Even if Garrett’s presumption (1996) is true, “that each denture wearer achieves a certain level of chewing performance (...) not markedly affected by the clinical quality of dentures,” chewing performance as the ability to comminute food is not the only objective criterion because an objective assessment can be gained from the perceived pain and discomfort accompanying masticatory load transfer. A further enhancement can be made with modelling of soft tissue nonlinear behavior, although the potential is more for scientific research, rather than for clinical practice as the robustness of calculation is the first demand.

The discovery of the mechanism of pain creation beneath a tilted denture, while being in balance with bilateral occlusal forces and the reaction force of the supporting zone (depending on the inclination of denture), explains the weak correlation between denture clinical scores and patient perceived comfort, and also shows why the occlusion settings based on the center of force in intercuspation (Kumar and D’Souza 2010), even if aided in vivo with computer force balance (Olivieri et al. 1998), have a poor association with mastication efficiency. Simultaneously, the decrease in the denture-supporting zone due to inclination (Żmudzki et al. 2012) shows that masticatory ability is not part of a simple relationship with foundation shape. Enlargement of the load-bearing area (i.e., contact area) beneath an inclined denture is the most beneficial factor in reducing pain; thus, the compliance of the denture soft lining material is important for improving the cushioning effect (Chladek et al. 2014; Saber-Sheikh et al. 1999). Additionally, destabilization under mastication loads is essential to better understand the impact of denture adhesives, that is, testing under a stable occlusion (Polyzois et al. 2014), as is the concept of a neutral zone and the development of rehabilitation exercises.

Objective assessment of mastication comfort is essential for the future investigation of the teeth’s optimal shape and position. These studies use excessively low-pressure values (Takayama et al. 2011; Arksornnukit et al. 2011) that are critical for ulcer formation but not for pain and do not provide quantifiable findings relating to the PPT (Koyano et al. 2012; Kumar 2014).

Moreover, the possibility of assessment of occlusal load distribution between artificial implantological supports and the mucosa opens the way for understanding the work of denture attachments and diminishing loads on the implants (Żmudzki et al. 2012b, 2013).
